# Nonsmoking and Nondrinking Oral Squamous Cell Carcinoma Patients: A Different Entity

**DOI:** 10.3389/fonc.2021.558320

**Published:** 2021-06-28

**Authors:** Zhan Yang, Wei Du, Xu Zhang, Defeng Chen, Qigen Fang, Yuezhong He, Yang Yang, Ding Li, Jie Fan

**Affiliations:** ^1^ Academy of Military Medical Sciences, Academy of Military Sciences, Beijing, China; ^2^ Department of Head Neck and Thyroid Surgery, Affiliated Cancer Hospital of Zhengzhou University, Henan Cancer Hospital, Zhengzhou, China; ^3^ Department of Nephrology, The First Affiliated Hospital of Zhengzhou University, Zhengzhou, China; ^4^ Department of Pharmacy, Affiliated Cancer Hospital of Zhengzhou University, Henan Cancer Hospital, Zhengzhou, China

**Keywords:** nonsmoking, nondrinking, HPV, head and neck squamous cell carcinoma, p16

## Abstract

**Objective:**

Our goal was to analyze the demographic and pathologic characteristics as well as prognosis in nonsmoking and nondrinking (NSND) oral squamous cell carcinoma (SCC) patients compared with typical oral SCC patients.

**Patients and Methods:**

A total of 353 patients were retrospectively enrolled and divided into two groups: the NSND group and the current smoking/current drinking (CSCD) group. Demographic, pathologic, and molecular data were compared between the two groups. The main research endpoints were locoregional control (LRC) and disease-specific survival (DSS).

**Results:**

In the NSND group, 16.3%, 41.9%, and 53.5% of patients were aged no more than 40 years, were female, and had an educational background of high school or above compared to 3.7%, 6.0%, and 38.2% of patients in the CSCD group, respectively. A total of 15.1% of the NSND patients had SCC of the lower gingiva and floor of the mouth, which was lower than the 35.6% of patients in the CSCD group. CSCD patients were likely to have an advanced disease stage (48.7% vs 32.5%, p=0.042) and poorly differentiated cancer (26.6% vs 16.3%, p=0.042). The NSND patients had a mean Ki-67 index of 24.5%, which was lower than the mean of 35.7% in the CSCD patients. The two groups had no HPV infection and similar p16 expression (4.7% vs 10.1%, p=0.132), but there was higher expression of p53 (38.6% vs 17.4%, p<0.001) and p63 (59.9% vs 29.1%, p<0.001) in the CSCD group. The 5-year LRC rates for NSND patients and CSCD patients were 48% and 38%, respectively, and the difference was significant (p=0.048). The 5-year DSS rates for NSND patients and CSCD patients were 56% and 39%, respectively, and the difference was significant (p=0.047). Further, a Cox model confirmed the independence of smoking and drinking status for affecting LRC and DSS.

**Conclusion:**

NSND oral SCC patients are a different entity. HPV infection has a limited role in carcinogenesis in NSND patients, and p16 expression is associated with worse locoregional control.

## Introduction

Oral squamous cell carcinoma (SCC) is the most common malignancy in cancers of the head and neck (1), and it significantly threatens people’s lives and quality of life. The latest epidemiologic data in 2011 showed that in China, the age-standardized incidence and mortality rates of oral SCC were 2.22 per 100,000 and 0.9 per 100,000, respectively (2). Tobacco smoking and alcohol consumption are considered to be the main risk factors and are responsible for at least 80% of oral SCC patients (3–5). There are 50 potential carcinogens including polycyclic aromatic hydrocarbons and nitrosamines in tobacco, and they can result in mutations of some important genes such as the tumor suppressor gene p53 that disturb modulation of the immune system and cell cycle regulation (6). The carcinogenic mechanism of alcohol is complex and might be involved in the genotoxic effects of acetaldehyde, genetic polymorphisms, cytochrome P450 2E1-mediated generation of reactive oxygen species, aberrant metabolism of folate and retinoids, and increased estrogen (7).

Although there has been increased knowledge regarding giving up smoking and drinking, the incidence of oral SCC has not decreased significantly (8, 9), and even nonsmoking and nondrinking (NSND) oral SCC patients are increasingly common. A number of previous researchers have tried to determine the difference regarding etiology, pathologic characteristics, and molecular expression as well as prognosis between nonsmoking patients and typical patients (10–14), but unfortunately, there is great controversy. Some authors have depicted that there is no significant survival difference between these two groups (10–12), some have reported that nonsmoking patients have a better prognosis (13), and some have described that there is worse survival in young nonsmoking patients (14). The majority of these studies did not limit their patients to NSND patients, and this minor designation flaw may not completely eliminate their potential confounding effects (1). On the other hand, literature on the molecular expression of NSND patients remains scarce, even though the reported rates of HPV16 infection, p16 expression, and p53 expression vary greatly (15–19). Therefore, in the current study, we aimed to analyze the demographic and pathologic characteristics as well as prognosis in NSND oral SCC patients compared with typical oral SCC patients.

## Patients and Methods

### Ethnic Consideration

Our Hospital institutional research committee approved our study, and all participants signed an informed consent agreement. All methods were performed in accordance with the relevant guidelines and regulations. All procedures performed in studies involving human participants were in accordance with the ethical standards of the institutional and/or national research committee and with the 1964 Helsinki Declaration and its later amendments or comparable ethical standards.

### Patient Selection

From January 2014 to December 2018, the medical records of 654 patients with surgically treated oral SCC were retrospectively reviewed. Oral SCC referred to SCC arising from the tongue; buccal, lower and upper gingiva, and the floor of the mouth. The included patients met the following criteria: the disease was primary; there was no history of other cancers; there was no habit of betel-nut chewing; the patient was classified as a NSND or a current smoker or current drinker (CSCD); and there was enough paraffin-embedded tissue available for HPV detection. Patients without sufficient demographic, pathologic, or follow-up data were excluded from the analysis. Information regarding age, sex, smoking, alcohol consumption, educational background, family cancer history, pathologic TNM stage (8^th^ AJCC system), pathologic reports, treatment, and follow-up was extracted and analyzed.

### Important Variable Definition

A NSND patient was defined as a patient who had smoked no more than 100 cigarettes and had simultaneously drank wine no more than once every two weeks in their lifetime (20–22). A CSCD patient was defined as a patient who had smoked at least 20 cigarettes per day for at least 10 years or had drank wine at least once per day for at least 10 years (14, 15, 19). All pathological sections were re-reviewed by at least two pathologists in a double-blind manner. Perineural invasion (PNI) was considered to be present if tumor cells were identified within the perineural space and/or nerve bundle; lymphovascular infiltration (LVI) was positive if tumor cells were noted within the lymphovascular channels (3, 23). Similar to our previous research (23), data on the family cancer history were obtained at initial treatment. During the preparation of this article, a questionnaire was sent to the patients or their family by email, postal letter, or WeChat if the information was not recorded clearly. The family members in the current study only consisted of first-degree relatives, and the patients were categorized as having a family cancer history if any of those relatives had any cancer other than nonmelanoma skin cancer. Otherwise, the patient was recorded as not having a family cancer history (23). The pathologic depth of invasion (DOI) was measured from the level of the adjacent normal mucosa to the deepest point of tumor infiltration, regardless of the presence or absence of ulceration (24).

### Immunohistochemical (IHC) Analysis

From July 2013, routine immunohistochemical analysis of Ki-67, p16, p53, and p63 was performed for every head and neck SCC patient. The level of positivity of p16 overexpression was consistent with previous studies (17, 19): 0-+, defined as less than 25% tumor staining; ++, defined as 25-50% tumor stating; +++, defined as 50-75% tumor staining; and ++++: defined as more than 75% tumor staining. Tumors with levels of +++ and ++++ were classified as having p16 positivity. Similar standards were used for p53 and p63. The Ki-67 score (0-100%) was calculated by the ratio of the number of immunostained nuclei to the total number of nuclei in tumor cells. The counting was performed in three randomly selected fields at ×400 magnification. The cut-off value of the Ki-67 score in the current study was defined as the median value (25, 26).

### HPV Assessment

From July 2013, HPV detection was selectively performed in fresh tumor tissue from oral SCC patients in our cancer center. DNA was extracted using the TIANcombi DNA Lyse&Det PCR Kit (TIANGEN Cooperation, Beijing, China) and was then subjected to real-time PCR with the INNO-LIPA HPV Genotyping Extra System^®^ kit (Innogenetics), which can detect 7 low-risk HPV types (6, 11, 40, 43, 44, 54, 70), 3 indeterminate-risk types (69, 71, 74), and 18 high-risk HPV types (16, 18, 26, 31, 33, 35, 39, 45, 51, 52, 53, 56, 58, 59, 66, 68, 73, 82). For paraffin-embedded tissue, at least five 10-µm thick slices were used for DNA extraction with the TIANcombi DNA Lyse&Det PCR Kit (TIANGEN Cooperation, Beijing, China) according to the instructions. The following procedures were similar to those described above.

### Surgical Principle

In our cancer center, systemic ultrasound, CT, MRI and/or PET-CT examinations were routinely performed for every patient. All oral SCC operations were performed under general anesthesia. The primary tumor was completely excised with at least a 1 cm margin; if necessary, a pedicled flap or free flap was used to close the defect. Neck dissection was usually performed except for tumors with very small sizes in the upper gingiva; levels of I to III were manipulated for a cN0 neck, and levels of I to IV or V were manipulated for a cN+ neck. Adjuvant treatment was suggested if T3/4 disease, cervical nodal metastasis, PNI, LVI, or positive margins were present.

### Statistical Analysis

Student’s *t* test was used to compare the continuous variables between the two groups, and the Chi-square test was used to compare the categorical variables between the two groups. The main study points were locoregional control (LRC) and disease-specific survival (DSS). The survival time of LRC was calculated from the date of surgery to the date of local, regional or locoregional recurrence or to the last follow-up, and the survival time of DSS was calculated from the date of surgery to the date of cancer-related death or the last follow-up. The Kaplan-Meier method (log-rank test) was used to calculate the LRC and DSS rates. The factors that were significant in univariate analysis were then analyzed in the Cox proportional risk regression model to determine the independent prognostic factors. All reported p values were two-sided, and a value of p<0.05 was considered significant. All statistical analyses were performed with SPSS 20.0.

## Results

### Demographic Characteristics

A total of 353 patients (301 males and 52 females) were enrolled for analysis. The NSND group consisted of 86 patients with a mean age of 50.6 (range: 30-68) years; 14 (16.3%) patients were aged ≤40 years, and there were 50 (58.1%) males and 36 (41.9%) females. Forty-six (53.5%) patients had an educational background of high school or above. Six (7.0%) patients had a family cancer history: esophageal cancer was noted in 4 (66.7%) families, and lung cancer was noted in the remaining two families (33.3%). The CSCD group consisted of 267 patients with a mean age of 62.5 (range: 38-76) years; 10 (3.7%) patients were aged ≤40 years, and there were 251 (94.0%) males and 16 (6.0%) females. A total of 102 (38.2%) patients had an educational background of high school or above. Twenty-nine (10.9%) patients had a family cancer history: esophageal cancer was noted in 13 (44.8%) families, lung cancer was noted in 7 (24.1%) families, breast cancer was noted in 4 (13.8%) families, liver cancer was noted in 3 (10.3%) families, and colorectal cancer was noted in 2 (6.9%) families. Patients in the NSND group were more likely to be female (p<0.001), have a younger age (p<0.001) and have a higher educational background (p=0.012) than those in the CSCD group. There were no apparent differences regarding family cancer history between the two groups (p=0.294) (**Table 1**).

**Table 1:** Comparison of demographic, pathologic, and molecular information between the non-smoker and non-drinker group (NSND) and the current-smoker/current-drinker (CSCD) group.

Variables	NSND group (n = 86)	CSCD group (n = 267)	p
Age			
≤40	14 (16.3%)	10 (3.7%)	
40-60	52 (60.4%)	95 (35.5%)	
≥60	20 (23.3%)	165 (61.8%)	<0.001
Sex			
Male	50 (58.1%)	251 (94.0%)	
Female	36 (41.9%)	16 (6.0%)	<0.001
Education background			
High school or above	46 (53.5%)	102 (38.2%)	
Under high school	40 (46.5%)	165 (61.8%)	0.012
A family cancer history			
Yes	6 (7.0%)	29 (10.9%)	
No	80 (93.0%)	238 (89.1%)	0.294
Primary tumor site			
Tongue	37 (43.0%)	89 (33.3%)	
Buccal	20 (23.3%)	57 (21.3%)	
Upper gingiva	16 (18.6%)	26 (9.7%)	
Lower gingiva	7 (8.1%%)	53 (19.9%)	
Floor of the mouth	6 (7.0%)	42 (15.7%)	0.005
Depth of invasion (mm)	8.2 (2.0-23.5)	9.9 (2.0-27.1)	<0.001
Pathologic tumor stage			
T1	19 (22.1%)	55 (20.6%)	
T2	39 (45.3%)	82 (30.7%)	
T3	18 (20.9%)	80 (30.0%)	
T4	10 (11.6%)	50 (18.7%)	0.042
Tumor differentiation			
Well	37 (43.0%)	80 (30.0%)	
Moderate	35 (40.7%)	116 (43.4%)	
Poor	14 (16.3%)	71 (26.6%)	0.042
Perineural invasion			
Positive	13 (15.1%)	65 (24.3%)	
Negative	73 (84.9%)	202 (75.7%)	0.073
Lymphovascular invasion			
Positive	12 (14.0%)	57 (21.3%)	
Negative	74 (86.0%)	210 (78.7%)	0.133
Pathologic neck stage*			
N0	45 (59.2%)	116 (46.0%)	
N1	20 (26.3%)	86 (34.1%)	
N2	11 (14.5%)	50 (19.8%)	0.130
Margin status			
Positive	6 (7.0%)	27 (10.1%)	
Negative	80 (93.0%)	240 (89.9%)	0.385
p16			
Positive	4 (4.7%)	27 (10.1%)	
Negative	82 (95.3%)	240 (89.9%)	0.132
p53			
Positive	15 (17.4%)	103 (38.6%)	
Negative	71 (82.6%)	164 (61.4%)	<0.001
p63			
Positive	25 (29.1%)	160 (59.9%)	
Negative	61 (70.9%)	107 (40.1%)	<0.001
Ki-67	24.5% (3.0%-78.5%)	35.7% (5.5%-93.0%)	<0.001

*Only patients who underwent neck dissection were analyzed.

### Operation and Pathologic Characteristics

In the NSND group, 15 (17.4%) patients underwent free flap reconstruction: 10 with radial forearm flaps, 3 with anterolateral flaps, and 2 with fibular flaps. Tongue SCC was present in 37 (43.0%) patients, buccal SCC was present in 20 (23.3%) patients, and SCC of the upper and lower gingiva was present in 16 (18.6%) and 7 (8.1%) patients, respectively. SCC in the floor of the mouth was present in 6 (7.0%) patients. The median DOI was 8.2 mm, with a range from 2.0 mm to 23.5 mm. The pathologic tumor stages were distributed as T1 in 19 (22.1%) patients, T2 in 39 (45.3%) patients, T3 in 18 (20.9%) patients, and T4 in 10 (11.6%) patients. Tumor differentiations of well, moderate, and poor were reported in 37 (43.0%), 35 (40.7%), and 14 (16.3%) patients, respectively. PNI and LVI were reported in 13 (15.1%) and 12 (14.0%) patients, respectively. Negative margins were achieved in 80 (93.0%) patients. Neck dissection was performed in 76 patients, and the pathologic neck lymph node stages were distributed as N0 in 45 (59.2%) patients, N1 in 20 (26.3%) patients, and N2 in 11 (14.5%) patients.

In the CSCD group, 61 (22.8%) patients underwent free flap reconstruction: 37 with radial forearm flaps, 9 with anterolateral flaps, and 15 with fibular flaps. Twenty (7.5%) patients underwent submental island flap reconstruction. Tongue SCC was present in 89 (33.3%) patients, buccal SCC was present in 57 (21.3%) patients, and SCC of the upper and lower gingiva was present in 26 (9.7%) and 53 (19.9%) patients, respectively. SCC in the floor of the mouth was present in 42 (15.7%) patients. The median DOI was 9.9 mm, with a range from 2.0 mm to 27.1 mm. The pathologic tumor stages were distributed as T1 in 55 (20.6%) patients, T2 in 82 (30.7%) patients, T3 in 80 (30.0%) patients, and T4 in 50 (18.7%) patients. Tumor differentiations of well, moderate, and poor were reported in 80 (30.0%), 116 (43.4%), and 71 (26.6%) patients, respectively. PNI and LVI were reported in 65 (24.3%) and 57 (21.3%) patients, respectively. Negative margins were achieved in 240 (89.9%) patients. Neck dissection was performed in 252 patients, and the pathologic neck lymph node stages were distributed as N0 in 116 (46.0%) patients, N1 in 86 (34.1%) patients, and N2 in 50 (19.8%) patients.

The two groups had significant differences regarding the primary tumor site (p=0.005), pathologic DOI (p<0.001), pathologic tumor stage (p=0.042), and tumor differentiation (p=0.042). Additionally, the two groups had a similar distribution of pathologic neck lymph node stage (p=0.130), PNI (p=0.073), and LVI (p=0.133) (**Table 1**).

#### HPV Infection, p16, p53, p63, and Ki-67

In the NSND group, no patients had HPV infection. Positivity of p53, p63, and p16 was reported in 15 (17.4%), 25 (29.1%), and 4 (4.7%) patients, respectively. The mean Ki-67 proliferation index was 24.5% (range: 3.0%-78.5%).

In the CSCD group, no patients had HPV infection. Positivity of p53, p63, and p16 was reported in 103 (38.6%), 160 (59.9%), and 27 (10.1%) patients, respectively. The mean Ki-67 proliferation index was 35.7% (range: 5.5%-93.0%).

Compared to the CSCD patients, the NSND patients had a significantly lower Ki-67 index (p<0.001). However, the CSCD patients had higher expression of p53 (p<0.001) and p63 (p<0.001). The two groups had similar distributions of p16 expression (p=0.132).

### Survival Analysis

During our follow-up with a median time of 34 months, in the NSND group, 45 patients received adjuvant radiotherapy, and 19 patients underwent adjuvant chemotherapy. A total of 37 patients suffered from disease recurrence: 34 cases locoregionally and 3 cases distantly. Only 10 patients were successfully salvaged by radical surgery. Nineteen patients died of the disease.

In the CSCD group, 162 patients received adjuvant radiotherapy, and 81 patients underwent adjuvant chemotherapy. A total of 150 patients suffered from disease recurrence: 141 cases locoregionally and 9 cases distantly. Only 40 patients were successfully salvaged by radical surgery. A total of 100 patients died of the disease.

**Figure 1:**
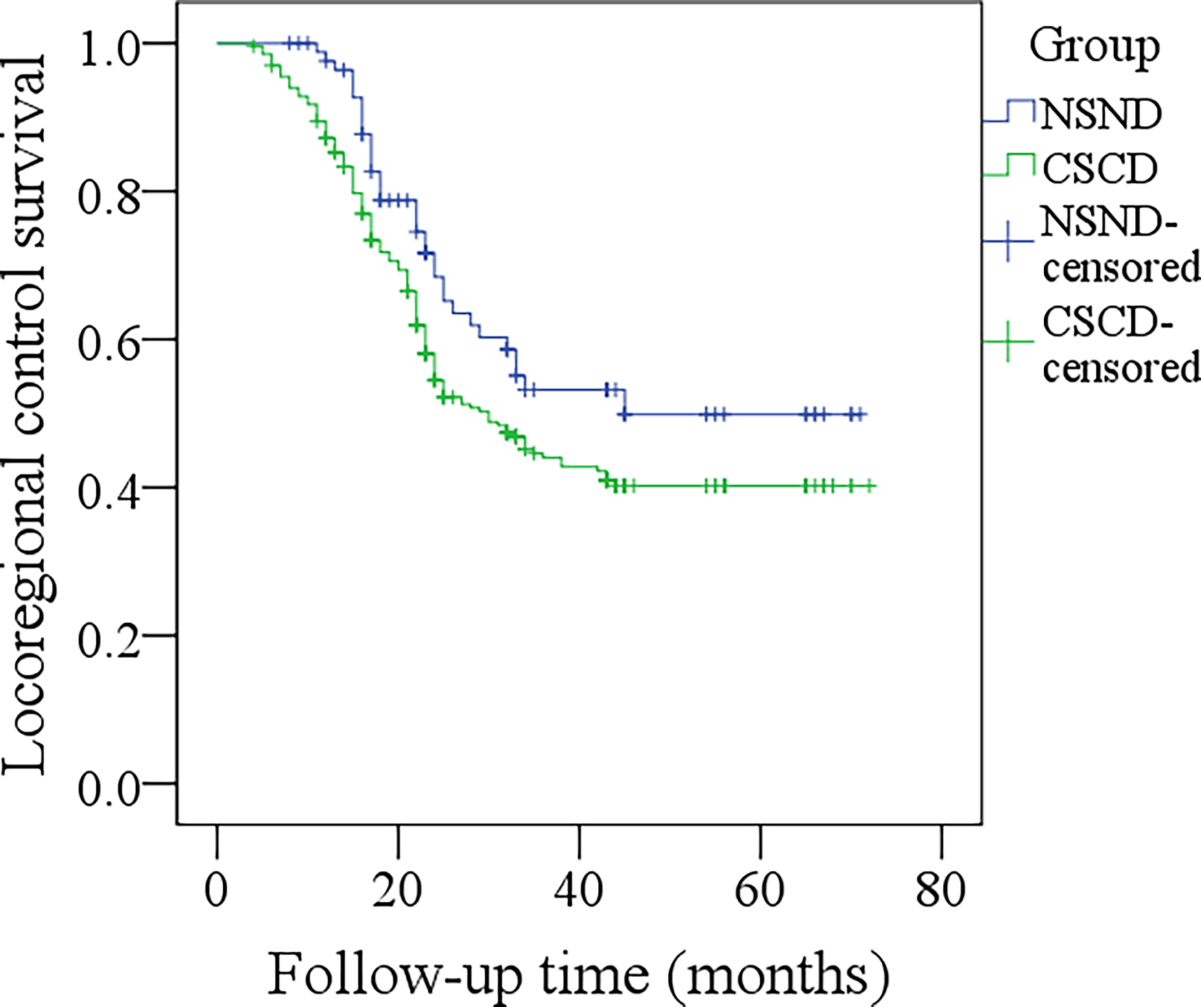
Comparison of locoregional control survival between the non-smoker and non-drinker group and the current-smoker or current-drinker (CSCD) group (p = 0.048).

The 5-year LRC rates for NSND patients and CSCD patients were 48% and 38%, respectively, and the difference was significant (**Figure 1**, p=0.048). Further, the Cox model confirmed the independence of smoking and drinking status for affecting LRC (p=0.022, **Table 2**).

**Table 2:** Univariate analysis and Cox model analysis of risk factors for locoregional recurrence in oral squamous cell carcinoma.

Variables	Univariate		Cox model	
	p	HR	95% CI	p
Age (≤40 *vs* >40)	0.036	2.465	0.337-7.543	0.754
Sex	0.224			
Education background	0.463			
Family cancer history	0.044	0.576	0.257-0.832	0.032
Tumor stage (T1+T2 *vs* T3+T4)	<0.001	6.563	2.341-18.427	<0.001
Tumor differentiation	<0.001			
Well				
Moderate		2.867	1.632-6.778	0.006
Poor		4.876	2.559-16.142	<0.001
Neck stage (N0 *vs* N+)	<0.002	5.337	1.863-19.226	<0.001
Perineural invasion	0.004	3.206	1.332-6.786	0.003
Lymphovascular invasion	0.012	5.789	0.116-30.321	0.554
Margin status	<0.001	5.216	1.632-18.331	<0.001
Status of smoking and drinking				
(NSND *vs* CSCD)	0.048	2.442	1.278-6.442	0.022
p16	0.036	2.335	1.327-7.002	0.019
p53	0.543			
p63	0.478			
Ki-67 (≤32.5% *vs* >32.5%)	<0.001	3.547	1.542-8.673	0.001
Adjuvant treatment	0.669			

**Figure 2:**
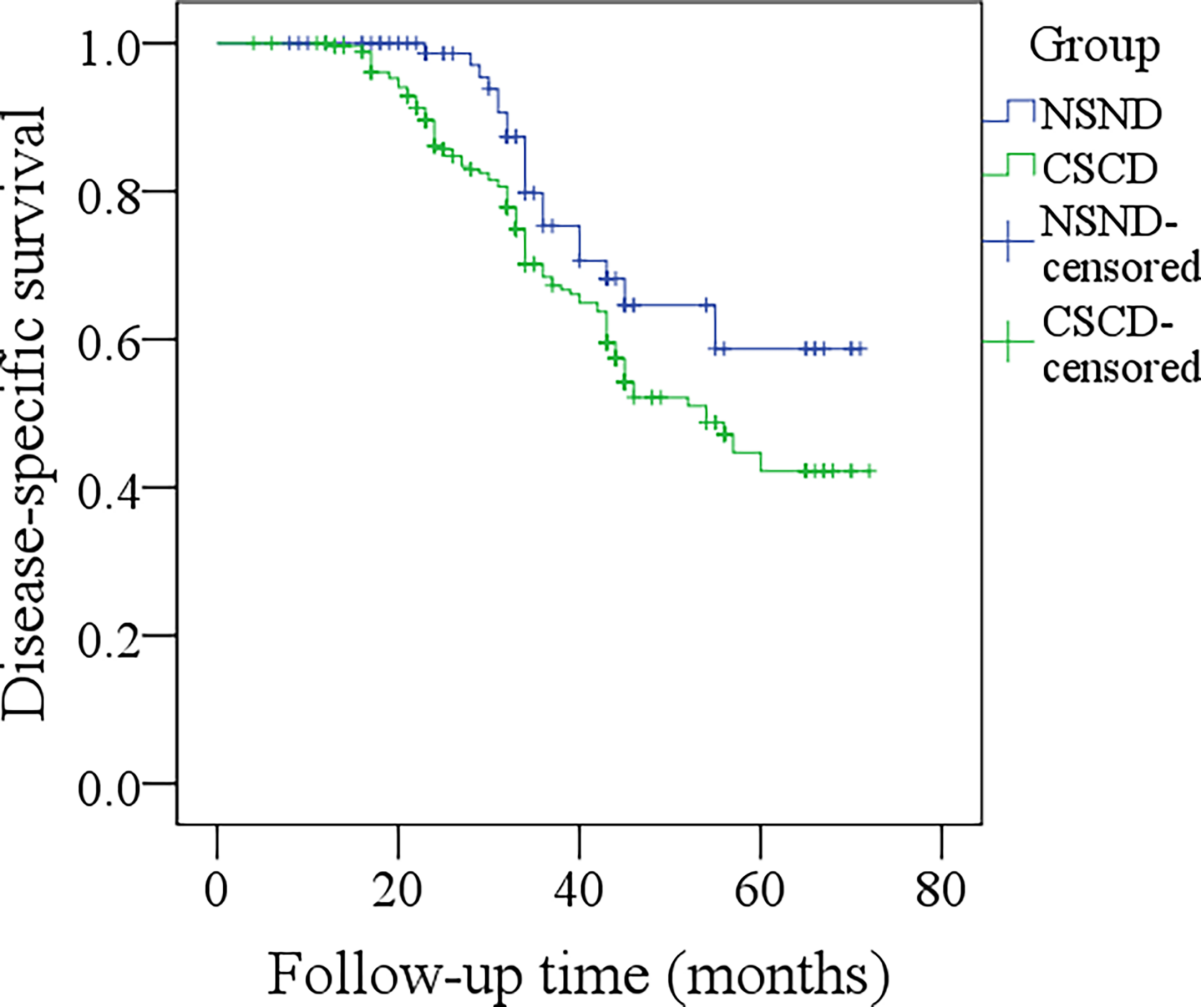
Comparison of disease-specific survival between the non-smoker and non-drinker group and the current-smoker or current-drinker (CSCD) group (p = 0.047).

The median DSS time for NSND patients and CSCD patients was 59.3 months and 54.0 months, respectively. The 5-year DSS rates for NSND patients and CSCD patients were 56% and 39%, respectively, and the difference was significant (**Figure 2**, p=0.047). Further, the Cox model confirmed the independence of smoking and drinking status for affecting DSS (p=0.015, **Table 3**).

**Table 3:** Univariate analysis and Cox model analysis of risk factors for cancer-caused death in oral squamous cell carcinoma.

Variables	Univariate		Cox model	
	p	HR	95% CI	p
Age (≤40 *vs* >40)	0.089			
Sex	0.546			
Education background	0.882			
Family cancer history	0.034	0.694	0.221-0.829	0.016
Tumor stage (T1+T2 *vs* T3+T4)	<0.001	7.322	2.005-21.563	<0.001
Tumor differentiation	<0.001			
Well				
Moderate		3.097	1.547-7.355	0.004
Poor		6.863	2.444-19.337	<0.001
Neck stage (N0 *vs* N+)	<0.001	5.442	1.476-13.356	<0.001
Perineural invasion	0.032	3.206	0.832-6.786	0.324
Lymphovascular invasion	0.031	4.761	0.976-30.321	0.067
Margin status	<0.001	4.224	1.355-13.217	<0.001
Status of smoking and drinking				
(NSND vs CSCD)	0.047	2.665	1.443-7.614	0.015
p16	0.077			
p53	0.431			
p63	0.785			
Ki-67 (≤32.5% *vs* >32.5%)	<0.001	2.632	0.775-9.435	0.101
Adjuvant treatment	0.338			

## Discussion

The most significant finding in the current study was that compared to typical oral SCC patients, NSND patients had significantly different epidemiological, pathologic, and molecular features and better prognosis, suggesting that NSND patients might be a different entity. This finding prompts more personalized cancer treatment for traditional and NSND oral SCC patients and more high-quality studies to clearly clarify the etiology of NSND patients.

In the beginning of preparing this research, one of the most important factors was to identify a clear definition of NSND and CSCD patients, which would improve the reliability of this study. Different definitions of never/current smokers and never/current drinkers have been described by previous authors (1, 11–15, 17–22), and it was noted that in most of those studies, an affirmative never drinker even had one drink once a week. Current evidence distinctly proves that alcohol consumption apparently increases the risk of oral SCC (27). More importantly, the association of alcohol consumption with the relative risk for developing cancer tends to be dose-dependent (14); therefore, we should make a stricter standard for NSND patients, such as the definition used in this research. On the other hand, a typical oral SCC patient is usually associated with heavy tobacco and alcohol use for 10 years or more (28), and a similar viewpoint has also been reported by Brennan et al. (6), Koch et al. (10), Farshadpour et al. (11), and Harris et al. (12). Therefore, to clearly determine the difference between NSND and CSCD groups and eliminate the influence of confounding factors, we identified a stricter standard for CSCD patients.

It was noted that there was a younger age in the NSND group, and a similar finding was also described by previous authors (9–11). However, literature regarding age distribution is scarce. There were significantly more patients aged less than 40 years in the NSND group. On the other hand, there was a male predominance in both groups but a significantly higher proportion of women in the NSND group in the current study; a similar finding was also noted by Bachar et al. (14) and Durr et al. (20). These two demographic findings might vaguely suggest that there are unknown factors explaining the occurrence of SCC in NSND patients; however, the influence caused by environmental tobacco cannot be ignored. Tan et al. (29) found that exposure to environmental tobacco in the home was always reported by elderly women with head and neck SCC, and men usually had a higher possibility of second-hand smoke exposure owing to their occupational nature (19).

Tumor site specificity has been demonstrated by a number of researchers (21, 30). Compared to CSCD patients, NSND patients had a lower possibility of developing SCC of the floor of the mouth and the lower gingiva but a higher possibility of developing SCC in the upper gingiva. It has been proposed that because of gravity dependence, pooling saliva containing alcohol/tobacco-derived carcinogens leads to an increased prevalence of cancer in the lower location of the oral cavity. A greater presence of adverse pathologic characteristics, including PNI, LVI, poor tumor differentiation, and advanced disease stage, has also been reported by previous authors (13, 14, 22), and similar findings were also noted by us. However, it is difficult to attribute this phenomenon to internal differences between the two groups because long-term alcohol and tobacco use can accelerate the development of cancer and change the biological behavior of disease (12).

The clarification of molecular expression variation was one of our main goals, as it would provide the strongest evidence for answering whether NSND patients are a different entity. Very few authors have performed similar analyses (17–19). Considerable attention has been given to the HPV virus owing to its possible etiological mechanism in head and neck SCC occurrence (28). Western researchers have even described HPV as being responsible for at least 70% of newly diagnosed cases of oropharynx SCC (31), but the role of HPV in inducing oral SCC remains unclear. Dediol et al. (17) reported that 27% of their NSND patients were HPV positive, but HPV detected by PCR did not distinguish whether HPV had been activated, and this finding did not support the causal relationship of HPV infection with tumorigenesis. Recent evidence by de Abreu et al. (32) showed that the frequency of high-risk HPV types in oral cavity SCC was very low and was less than 4%, and the authors concluded that HPV was not involved in the genesis of oral cavity SCC. Our study would also support this viewpoint, as no HPV infection occurred in either groups.

Furthermore, p16 is usually evaluated together with HPV. For oropharynx SCC, there is a reliable association between HPV infection and p16 overexpression, and p16-IHC is usually regarded as a surrogate marker of HPV infection. However, in the current study, we noted that approximately 5% of the NSND patients showed p16 positivity, although no HPV infection was detected by PCR. In a previous report by Harris et al. (12), 40% of young oral tongue SCC patients had p16 positivity, but no HPV was found in any of the tumor samples. Similar findings were also noted by Poling et al. (33): 9 of the 78 patients had p16 positivity, but only 1 patient had HPV E6/E7 mRNA transcripts. Moreover, our two groups had similar distributions of p16 expression. These findings suggest that p16 is not suitable for assessing the etiology associated with HPV infection in oral SCC.

In addition, p53 and p63 have been widely analyzed in head and neck SCC, but only a few authors have analyzed their expression in NSND patients. Heaton et al. (18) reported that a total of 16 tumors had strong p53 expression with a prevalence of 31.4%, and a previous review depicted that the overall rate of p53 positivity in head and neck SCC varied from 20% to 90% (33), which was slightly higher than that (17.4%) in our NSND patients but was consistent with that in our CSCD patients. The variation was attributed to the fact that both tobacco and alcohol could lead to mutations in the TP 53 gene. p63 was rarely assessed in NSND patients, and we might be the first to report that 29.1% of NSND patients show strong expression of p63. Previous studies have shown that the expression of p63 in SCC tissue is significantly higher than that in epithelial dysplasia and normal tissues (34). Together with our findings, these results suggest a role for p63 expression in carcinogenesis, and the effect might be enhanced by tobacco and alcohol. Ki-67 is an indicator of cancer cell proliferation, and a greater Ki-67 index might indicate more aggressive and poorer disease survival (26). We might be the first to report that the mean Ki-67 proliferation index was 24.5% for NSND patients, which was significantly lower than that in typical patients. This finding again provides evidence that NSND patients might be a different entity.

Survival differences between NSND patients and CSCD patients have been frequently compared, and conflicting results have been reported. Bachar et al. (14) divided 291 patients into two groups based on the status of tobacco smoking and alcohol abuse, and the two groups had similar local and regional control rates as well as overall survival rates. However, Durr et al. (20) described that compared to former or current smoking patients, never smoking patients tended to have decreased overall survival. In our opinion, long-term exposure to tobacco and alcohol is linked to a higher risk of peripheral vascular disease, chronic obstructive pulmonary disease, and coronary artery disease. Therefore, the index of overall survival might not be reliable enough for detecting the survival difference between the two groups. Pytynia et al. (13) found that after being matched to 50 ever smokers according to important variables, never smokers had a greater DSS and recurrence-free survival, and a further Cox model confirmed its independence. Our previous study also suggested that smoking was associated with an approximately 2-fold increase in the risk for recurrence and a 5-fold increase in the risk for disease-related death (22). In the current study, we noted that compared to CSCD patients, NSND patients had significantly better LRC and DSS in both univariate and multivariate analyses. A similar finding was also reported by Farshadpour et al. (11). Thus, NSND oral SCC patients might be a different entity.

It was interesting to find the negative prognostic significance of p16 expression in oral SCC. As usual, p16 expression was related to better survival in oropharynx SCC, but the exact opposite result was found in oral SCC. In a recent publication by Dediol et al. (17), the authors also reported that p16 expression carried a negative prognosis in oral SCC patients. However, in a recent meta-analysis, Almangush et al. (35) noted that there was no sufficient evidence to support p53, Ki-67 and p16 as prognostic biomarkers for oral SCC. The prognostic significance of p63 in oral SCC remains unknown, and our study failed to report a significant relationship between p63 expression and survival. However, Xu-Monette et al. (36) described the protective effect of p63 expression in high-risk diffuse large B-cell lymphoma. Therefore, more high-quality studies are needed to clarify these questions.

The limitations of the current study must be stated: there was inherent bias within this retrospective study, which may have decreased our statistical power; some other potential risk factors including chronic periodontitis, oral hygiene and economic status were not taken into consideration; and our strict standard may have artificially widened the difference between the two groups.

## Conclusions

In summary, NSND oral SCC patients are a different entity compared with typical patients. HPV infection has a limited role in carcinogenesis in NSND, and p16 expression is associated with worse locoregional control.

## Data Availability Statement

All data generated or analyzed during this study are included in this published article; the primary data can be obtained from the corresponding authors.

## Ethics Statement

The Zhengzhou University institutional research committee approved our study, and all participants signed an informed consent agreement.

## Author Contributions

All authors listed have made a substantial, direct, and intellectual contribution to the work, and approved it for publication.

## Conflict of Interest

The authors declare that the research was conducted in the absence of any commercial or financial relationships that could be construed as a potential conflict of interest.

The reviewer XQ declared a shared affiliation, with no collaboration, with the authors to the handling editor at the time of review.
